# Optimization of head and neck vascular CT angiography using variable rate bolus tracking technique and third-generation dual-source CT dual-energy scanning

**DOI:** 10.1186/s12880-025-01613-4

**Published:** 2025-03-04

**Authors:** Wei-Hua Lin, Fei-Peng Zhang, Bing-Quan Wang, Rui-Gang Huang, A-Lai Zhan, Hui-Jun Xiao

**Affiliations:** Department of Radiology, Zhangzhou Affiliated Hospital of Fujian Medical University, Zhangzhou, China

**Keywords:** Head and neck vascular CTA, Bolus tracking technique, Variable rate, Contrast agent dosage, Dual-source CT

## Abstract

**Objective:**

To evaluate the effectiveness of the variable rate bolus tracking technique combined with third-generation dual-source CT dual-energy scanning in enhancing the quality of head and neck vascular CT angiography (CTA).

**Methods:**

We conducted a retrospective analysis of 202 patients who underwent head and neck vascular CTA using a third-generation dual-source CT with dual-energy scanning. Patients were divided based on the contrast injection method into two groups: the variable-rate bolus tracking group (Group A, *n* = 100) and the fixed flow rate group (Group B, *n* = 102). We compared subjective image quality, venous artifacts, and objective image quality parameters between the two groups.

**Results:**

The amount of contrast agent used in Group A was significantly lower than in Group B. Additionally, mean attenuation values of arterial segments in Group A were markedly lower than those in Group B. Compared to Group B, attenuation values of the intracranial venous sinuses, right jugular vein, superior vena cava, right subclavian vein, and left jugular vein in Group A showed significant reductions. No significant difference was observed in the subjective image quality between the two groups. However, venous artifact in the right subclavian vein was significantly diminished in Group A.

**Conclusion:**

The application of the variable rate bolus tracking technique alongside third-generation dual-source CT dual-energy scanning in head and neck vascular CTA can achieve high-quality imaging while reducing contrast agent dosage. It enhances the attenuation contrast of intracranial arteries and veins and minimizes residual contrast and artifacts in the right subclavian vein.

## Introduction

Computed tomography angiography (CTA) is pivotal for diagnosing vascular diseases in the head and neck, including conditions such as cerebral arterial and jugular venous malformations, stenosis, occlusion, and aneurysms [1, 2]. However, CTA poses risks, notably contrast-induced nephropathy (CIN) [3, 4], which has a reported prevalence ranging widely from 4.1 to 26.6% according to studies by Hossain et al. [5]. Minimizing the dosage of contrast agents is a critical focus in CTA research due to these potential adverse effects. Currently, a traditional injection scheme using a single flow rate for contrast medium, typically ranging from 40 to 100 ml, remains prevalent in clinical practice for head and neck CTA [6]. This method, however, faces challenges due to the rapid circulation within intracranial arteries and veins, where the peak difference between the two is a mere 6–8 s, leading to venous interference during arterial imaging [7]. Furthermore, residual contrast in the subclavian vein and superior vena cava can significantly degrade the imaging quality of crucial areas like the openings of the common carotid and vertebral arteries.

Segmented variable rate bolus tracking technology leverages bolus tracking by adjusting the contrast agent injection flow rate. This technique extends the duration of vascular enhancement, thereby optimizing imaging quality and providing better depiction of lesions or vascular structures. Additionally, the low kiloelectron volt (keV) images produced by the dual-energy scanning mode of the third-generation dual-source CT can enhance CTA imaging of the head and neck, lungs, coronary arteries, and lower limbs, thus achieving optimal vessel enhancement with reduced contrast agent dosages [8–11]. The dual-energy bone removal software further simplifies the process by eliminating bone and calcification interference on head and neck artery images, offering a more straightforward approach than the dual-scan subtraction bone removal used in single-source CT.

This study aims to leverage variable rate bolus tracking technology in combination with third-generation dual-source CT dual-energy scanning to produce high-quality images for head and neck vascular CTA while simultaneously reducing the required dosage of contrast agents.

## Materials and methods

This retrospective study received approval from the ethics review committee of Fujian Medical University Affiliated Zhangzhou City Hospital. It included patients who underwent head and neck vascular CTA examinations. Exclusion criteria were established to ensure patient safety and image quality, including allergies to iodinated contrast agents, severe liver dysfunction defined as Child-Pugh grade C; [12] and kidney dysfunction defined as an estimated glomerular filtration rate (eGFR) of less than 30 mL/min/1.73 m²; [13] inability to cooperate with the examination procedures; inability to lie in a supine position; pregnancy; weight exceeding 90 kg; age under 18 years; acute increase in intracranial pressure; cerebral hemorrhage; severe heart failure; significant motion artifact during imaging; severe cerebral and cervical stenosis; absence of venous access in the right upper limb; patients with subclavian vein catheterization; and jugular vein reflux.

Patient clinical information, such as gender, age, and weight, was retrieved from the electronic medical record system. Data on the examination method, contrast agent dosage, and radiological findings were collected from the imaging system. Serum creatinine levels before and after the CTA examination (the highest serum creatinine level recorded between 48 and 72 h post-examination) were gathered via the laboratory information system.

Adhering to predetermined inclusion and exclusion criteria, data for 202 patients were compiled from the hospital’s electronic medical record system for the period between July 2022 and April 2023. The patients were divided into two groups based on the sequence of examination and the technique of contrast agent injection: Group A consisted of 100 patients using a variable rate bolus tracking injection method, while Group B comprised 102 patients utilizing a traditional single rate bolus tracking technique. A dedicated team of technicians and physicians conducted the study, ensuring that the examination methodologies and procedures were thoroughly communicated to the patients and their families.

Renal function was assessed in both groups prior to and following the examination. The diagnostic criterion for post-contrast acute kidney injury (PC-AKI), formerly recognized as CIN, was determined by an increase in serum creatinine of ≥ 0.3 mg/dL (≥ 26.5 μmol/L) or an increase to 1.5–1.9 times from the baseline value within 48–72 h after the use of a contrast agent [5]. 

### Equipment and parameters

Both groups underwent a standardized head and neck CTA examination using a third-generation dual-source CT scanner (SOMATOM Force, Siemens Healthineers, Forchheim, Germany). The scanning protocol included anteroposterior and lateral positioning images spanning from the vertex to the lower edge of the heart. The timing for the scan initiation was determined by employing bolus tracking technology. Bolus tracking scanning parameters were set as follows: the monitoring level was positioned near the ascending aorta at the level of the aortic arch, with a tube voltage of 100 kVp and a fixed tube current of 23 mAs. The trigger threshold for the start of the scan differed between the two groups; Group A utilized variable rate bolus tracking technology with a lower iodine flow rate, setting the trigger threshold at 60 HU, while Group B’s trigger threshold was set at 100 HU. For dual-energy scanning, the parameters included a foot-to-head scanning direction, covering the range from the lower edge of the tracheal bifurcation to the vertex. The delay was set at 3 s after reaching the trigger threshold, employing a dual-energy scanning mode with tube voltages of 80/sn150 kVp. The acquisition was performed with a configuration of 192 × 0.6 mm, a pitch of 0.7, and a rotation time of 0.25 s. The organ characteristic was set to NECK, with quality reference mAs of 129 mA for 80 kV and 72 mA for sn150 kVp, and CARE Dose 4D was activated for dose optimization. Reconstruction parameters included a slice thickness of 0.75 mm, an increment of 0.5 mm, a field of view (FOV) that encompassed the entire skull, and a matrix size of 512 × 512. The reconstruction kernel used was Qr40 with a CT angiography window setting. The series was composed of A + B, with DE Comp set to 0.5 and the strength of ADMIRE set to 3, facilitating enhanced image quality and reduced noise.

### Contrast agent injection scheme

An overall view injector XD200x (Ulrich Medical, Germany) high-pressure injector was employed for contrast agent administration, using an 20G indwelling needle for venipuncture via the right median cubital vein. The contrast agent, Ioversol with a concentration of 350 mg I/ml, was provided by Jiangsu Hengrui Medicine Co., Ltd., China. To account for the influence of body weight on vascular enhancement, the flow rate of the contrast agent was adjusted according to the patient’s body weight [14, 15]. Specifically, for body weights ranging from 40 to 49 kg, 50–64 kg, 65–79 kg, to 80–90 kg, flow rates were set at 4.5 ml/s, 5 ml/s, 5.5 ml/s, and 6 ml/s, respectively.

Both groups utilized an intelligent tracking-triggered contrast agent injection method. Group A implemented a variable rate bolus tracking injection approach, consisting of a first phase with a flow rate of 2 ml/s for 3 s. In the second phase, the flow rate was adjusted based on the patient’s weight, with the contrast agent injected for 4 s. The third phase continued with a saline flush at the same flow rate as the second phase, extending for 10 s. Conversely, Group B adhered to a conventional single rate bolus tracking injection scheme, where the patient’s weight determined the flow rate. This involved an 8-second injection of the contrast agent in the first phase, followed by a saline flush at the same rate for 10 s in the second phase. The injection of the contrast agent in both schemes was continuous, with no interruption between phases. The monitoring scan started 8 s after the initiation of the contrast agent injection.

### Image quality evaluation

Upon completion of the examination, the images were transferred to a Siemens post-processing workstation (syngo.via, version VB40B, Siemens Healthineers, Forchheim, Germany). Here, the dual-energy software created linear blending (LB) automatic bone removal data, utilizing a fusion coefficient (M) of 0.5. This process enabled the direct generation of volume rendering (VR) images from the dataset without the need for manual adjustments, such as trimming or region growing. The images underwent a 360° clockwise rotation in 10° increments, yielding a total of 36 images. Curved planar reconstruction (CPR) images were also produced for the left and right internal carotid arteries and the left and right vertebral arteries using the non-bone-removed thin-layer images.

During the analytical phase, the vessels’ central line in the subjects was rotated at 0°, 45°, 90°, and 135° angles to assess the branching vessel stenosis from multiple perspectives. The NASCET criteria were used to evaluate vascular stenosis [16]. Moreover, for vascular segments with lesions, a specific angle was selected to better showcase the characteristics of the vascular lesions. This particular angle was determined based on the course of the vessels and the distribution of the lesions, ensuring a comprehensive display of the anatomical structure and the full extent of the lesion site for both subjective and objective evaluations.

Objective evaluation indicators on the workstation were determined by an experienced and professionally trained radiology technician responsible for objectively assessing the images for both groups. Main measurement indicators were the CT attenuation values of various branch vessels, including: (a) the ascending aorta at the level of the tracheal bifurcation, (b) bilateral common carotid arteries at their bifurcations, (c) bilateral middle cerebral arteries (M1 segment), and for the veins: (a) intracranial venous sinus confluence at the M1 segment level of the middle cerebral artery, (b) bilateral jugular veins at the common carotid arteries’ bifurcation, (c) superior vena cava at the starting level, and (d) right subclavian vein at its maximum diameter. Measurements were taken avoiding plaques and stenotic areas, focusing on the lumen’s center and measuring an area about half the lumen size for optimal results. Each measurement point was measured twice, averaging the two as the final result to ensure data measurement accuracy.

**Fig. 1:**
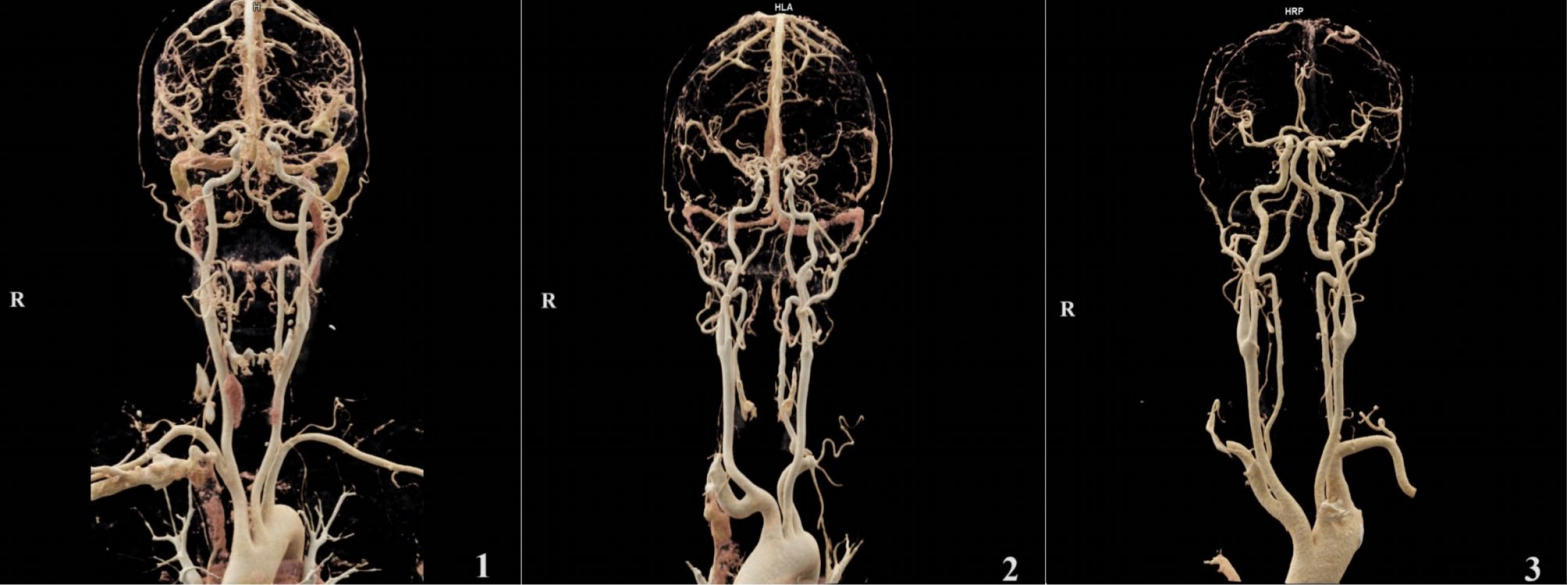
Schematic diagram of image quality scoring using a 5-point subjective rating scale. “**1**” is a 3-point image, “**2**” is a 4-point image, and “**3**” is a 5-point image

**Fig. 2:**
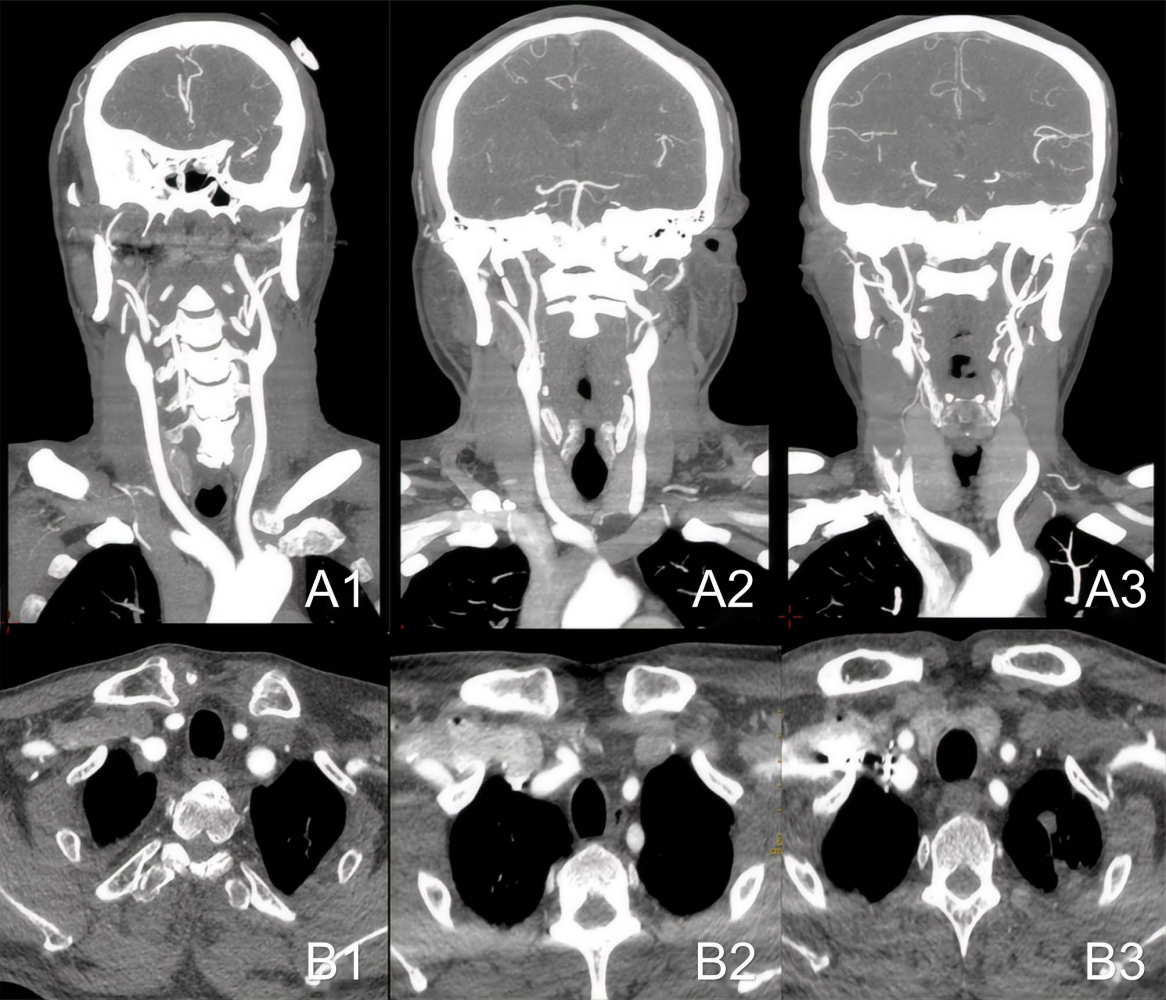
Grading diagram of contrast agent residue and artifacts in the right subclavian vein using a 4-level grading system. A is the coronal maximum intensity projection (MIP). “**A1**” is level 1, “**A2**” is level 2, “**A3**” is level 3, and “**B1-B3**” correspond to their respective transverse axial images

Subjective scoring: two head and neck radiologists, each with over ten years of experience, would independently evaluate the quality of both original and reconstructed images in a double-blind manner. The assessment of image quality is based on a 5-point subjective scoring criterion [17]. Scores of ≥ 3 points are deemed to satisfy clinical diagnostic requirements, 4 points indicate good diagnostic quality, and 5 points denote excellent image quality. (Fig. 1) Additionally, the evaluation of residual contrast agent and artifacts in the right subclavian vein employs a 4-level scoring system [17]. (Fig. 2) The specific criteria for both assessments are detailed in Tables 1 and 2, respectively.

**Table 1:** 5-point subjective scoring criteria

Score	Criteria	Description				
		Image contrast	Vessel edges	Vessels and branches	Artifacts	Diagnosis
1	Very poor	Low	Blurred	Main and branch vessels unrecognizable.	Severe	Affecting
2	Poor	Poor	Slight rough	Main vessels recognizable but branches difficult to display.	Noticeable	Possibly affecting
3	Average	Acceptable	Relatively clear	Main vessels and some branches visible.	Mild	Not affecting
4	Good	Better	Clear	Main vessels and branches identifiable.	Fewer	Not affecting
5	Excellent	Excellent	Very clear	Main vessels and all branches Visible	No	Meeting clinical requirements

**Table 2:** Level criteria description

Score	Criteria
1st Level	No high-concentration contrast agent residue in the vein.
2nd Level	Only a small amount of layered contrast agent residue in the subclavian vein lumen, accompanied by mild beam hardening artifacts, but no impact on arterial angiography.
3rd Level	Contrast agent retained in the entire vessel, moderate beam hardening artifacts present, but still meeting clinical diagnostic requirements.
4th Level	complete retention of the contrast agent in the entire vein, accompanied by severe beam hardening artifacts, affecting local diagnostic accuracy.

### Statistical methods

Experimental data were processed and analyzed utilizing Excel (Microsoft Corp, Redmond, WA) and SPSS version 23.0 (IBM SPSS Inc, Armonk, NY). Continuous variables that conformed to a normal distribution were presented as mean ± standard deviation and were subject to analysis via grouped t-tests. Categorical variables were represented as frequencies (percentages) and analyzed with chi-square tests. For data deviating from normal distribution, values were summarized as median [interquartile range] and examined using the nonparametric Wilcoxon test. The agreement between the subjective image quality scores assigned by the two radiologists was evaluated using the Kappa test. The kappa value interpretation was categorized as follows: κ < 0.40 indicated poor agreement; 0.40 ≤ κ < 0.75 signified moderate agreement; κ ≥ 0.75 represented good agreement. A *p*-value less than 0.05 was considered indicative of a statistically significant difference.

## Results

A total of 202 patients were enrolled in this study, with 100 patients in Group A and 102 in Group B. We evaluated gender, age, and weight across the groups, finding no significant statistical differences in these characteristics (*P* > 0.05), ensuring comparability of the study subjects on these basic metrics. The CTA examinations were completed without any adverse reactions reported. Furthermore, we analyzed the contrast agent doses utilized for the patients. The results demonstrated that the dose of contrast agent in Group A was reduced by 35% compared to Group B (*P* < 0.001) (Table 3). Before the examination, average serum creatinine levels were 69.4 ± 15.1μmol/L in Group A and 65.8 ± 11.2μmol/L in Group B. These levels adjusted to 71.3 ± 16.0μmol/L and 72.4 ± 15.4μmol/L, respectively, 48–72 h post-examination, with no significant differences observed (*P* > 0.05). Following the examination, no CIN occurred in either group. (Table 4)

**Table 3:** Comparison of clinical characteristics and contrast agent doses between two groups

Variable	Group A (*n* = 100)	Group B (*n* = 102)	*P*
Gender, n (%)			0.364
Male	66 (66.0%)	60 (58.8%)	
Female	34 (34.0%)	42 (41.2%)	
Age, median [range], y	61.0 [55.0;70.0]	63.0 [55.0;69.0]	0.671
Weight, median [range], kg	62.5 [56.0;69.2]	64.0 [56.2;69.0]	0.784
Contrast agent doses, median [range], ml	26.0 [26.0;28.0]	40.0 [40.0;44.0]	0.001

**Table 4:** Comparison of renal function pre- and post-examination across both groups

Progect	Group A (*n* = 100)	Group B (*n* = 102)	*P*
Pre-examination Scr	69.4 ± 15.1	65.8 ± 11.2	0.058
post-examination Scr *	71.3 ± 16.0	72.4 ± 15.4	0.603
CIN or PC-AKI	0	0	0

*Maximum serum creatinine level recorded between 48–72 h post-CTA examination

Objective image quality evaluation encompassed a total of 1010 arterial segments (202 aortic arches and 808 common carotid arteries- middle cerebral arteries) from 202 patients. Additionally, analyses included 202 right subclavian vein segments, 202 superior vena cava segments, 404 jugular vein segments, and 202 superior sagittal sinuses. The comparison of arterial attenuation values between the two groups revealed significant differences: ascending aorta CT values at the aortic arch plane were 357 ± 35.7 in Group A versus 411 ± 52.2 in Group B (*P* < 0.001); right carotid bifurcation CT values were 375 ± 39.0 in Group A versus 451 ± 63.6 in Group B (*P* < 0.001); left carotid bifurcation CT values were 375 ± 40.1 in Group A versus 458 ± 62.8 in Group B (*P* = 0.013); right middle cerebral artery M1 segment CT values were 323 [308; 343] in Group A versus 390 [357; 432] in Group B (*P* < 0.001); left middle cerebral artery M1 segment CT values were 321 [307; 344] in Group A versus 389 [357; 433] in Group B (*P* < 0.001), indicating lower arterial segment enhancements in Group A compared to Group B (Table 5).

**Table 5:** Comparison of image quality in the two groups

			Group A (*n* = 100)		Group B (*n* = 102)		*P*
**Subjective score**	κ	score	a n(%)	b n(%)	a n(%)	b n(%)	
5-point subjective scoring criterion*	0.775						0.9114
		3	2 (2.00)	3 (3)	2 (2.0)	2 (2)	
		4	20 (20.0)	19 (19)	18 (17.6)	15 (14.7)	
		5	78 (78.0)	78 (78)	82 (80.4)	85 (83.3)	
4-level scoring system^#^	0.871						< 0.001
		1	60 (60.0)	56 (56.0)	27 (26.5)	27 (26.5)	
		2	33 (33.0)	37 (37.0)	60 (58.8)	58 (56.9)	
		3	7 (7.00)	7 (7.00)	15 (14.7)	17 (16.7)	
**Objective evaluation**			CT values: median (range) or mean (SD)				
Venous sinus			112 [93.3;143]		153[122;182]		< 0.001
Jugular vein bifurcation of the LCCA			57.8 [48.5;65.0]		60.9[53.7;71.2]		< 0.001
Jugular vein bifurcation of the RCCA			59.0 [51.9;66.7]		63.7 [57.1;75.6]		< 0.001
Right subclavian vein			146 [98.0;209]		258 [187;386]		< 0.001
Superior vena cava			79.5 [66.8;100]		118 [96.2;149]		< 0.001
Arcus aortae			357 ± 35.7		411 ± 52.2		< 0.001
Bifurcation of LCCA			375 ± 40.1		458 ± 62.8		0.013
Bifurcation of RCCA			375 ± 39.0		451 ± 63.6		< 0.001
Left MCA M1 segment			321 [307;344]		389 [357;433]		< 0.001
Right MCA M1 segment			323 [308;343]		390 [357;432]		< 0.001

* Subjective score of image quality of original and reconstructed images. ^#^ Contrast retention and artifacts in right subclavian vein. LCCA: left common carotid artery, RCCA: right common carotid artery, MCA: middle cerebral artery

**Fig. 3:**
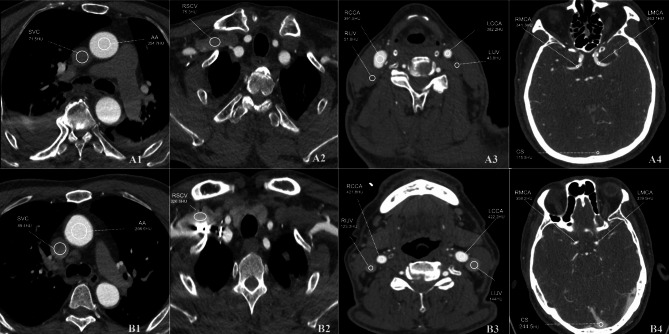
**Group A**: The different region of interest(ROI) of a patient with an image quality score of 5. Figures A1-4: transverse axial slices with a mixing coefficient of 0.5. **A1**: CT values - aorta (AA) was: 354.7 HU; superior vena cava (SVC): 71.5 HU. **A2**: right subclavian vein (RSCV): 75.3 HU. **A3**: common carotid arteries - left (LCCA): 382.2 HU, right (RCCA): 391.5 HU; internal jugular veins - left (LIJV): 43.8 HU, right (RIJV): 51.8 HU. **A4**: middle cerebral artery, M1 segment - left: 363.1 HU, right: 341.3 HU; sinus confluence (CS): 115.5 HU. **Group B**: The different ROI of a patient with an image quality score of 3. Figures B1-4: transverse axial thin sections with a mixing coefficient of 0.5. **B1**: CT values - AA: 396.9 HU; SVC: 89.4 HU. **B2**: RSCV: 326.6 HU. **B3**: LCCA: 422.2 HU, RCCA: 421.8 HU; LIJV: 144 HU, RIJV: 123.8 HU. **B4**: middle cerebral artery, M1 segment - Left: 339.5 HU, Right: 358.2 HU; CS: 244.5 HU

Venous segment attenuation comparisons between the two groups showed: superior sagittal sinus attenuation values were 112 [93.3; 143] in Group A versus 153 [122; 182] in Group B (*P* < 0.001); left jugular vein attenuation values were 57.8 [48.5; 65.0] in Group A versus 60.9 [53.7; 71.2] in Group B (*P* < 0.001); right jugular vein attenuation values were 59.0 [51.9; 66.7] in Group A versus 63.7 [57.1; 75.6] in Group B (*P* < 0.001); superior vena cava attenuation values were 79.5 [66.8; 100] in Group A versus 118 [96.2; 149] in Group B (*P* < 0.001); right subclavian vein attenuation values were 146 [98.0; 209] in Group A versus 258 [187; 386] in Group B (*P* < 0.001), with venous segment enhancements in Group A significantly lower than those in Group B, especially for the superior vena cava and subclavian vein where attenuation values in Group A were reduced by 32.6% and 43.4%, respectively. (Fig. 3)

The subjective image quality evaluation by two head and neck radiologists showed good agreement (kappa = 0.775), with all raw and reconstructed images deemed of high quality and suitable for clinical diagnosis. In Group A, 78% of images received 5-point scores, 20% received 4-point scores, and 2% received 3-point scores, with slight variations in the second assessment (78% for 5 points, 19% for 4 points, and 3% for 3 points). Group B saw 80.4% of images scoring 5 points and 83.3% in the second assessment, 17.6% scoring 4 points and 14.7% in the second assessment, and 2% scoring 3 points consistently in both assessments. The differences in subjective image quality scores between groups A and B were not statistically significant (*P* > 0.05).

**Fig. 4:**
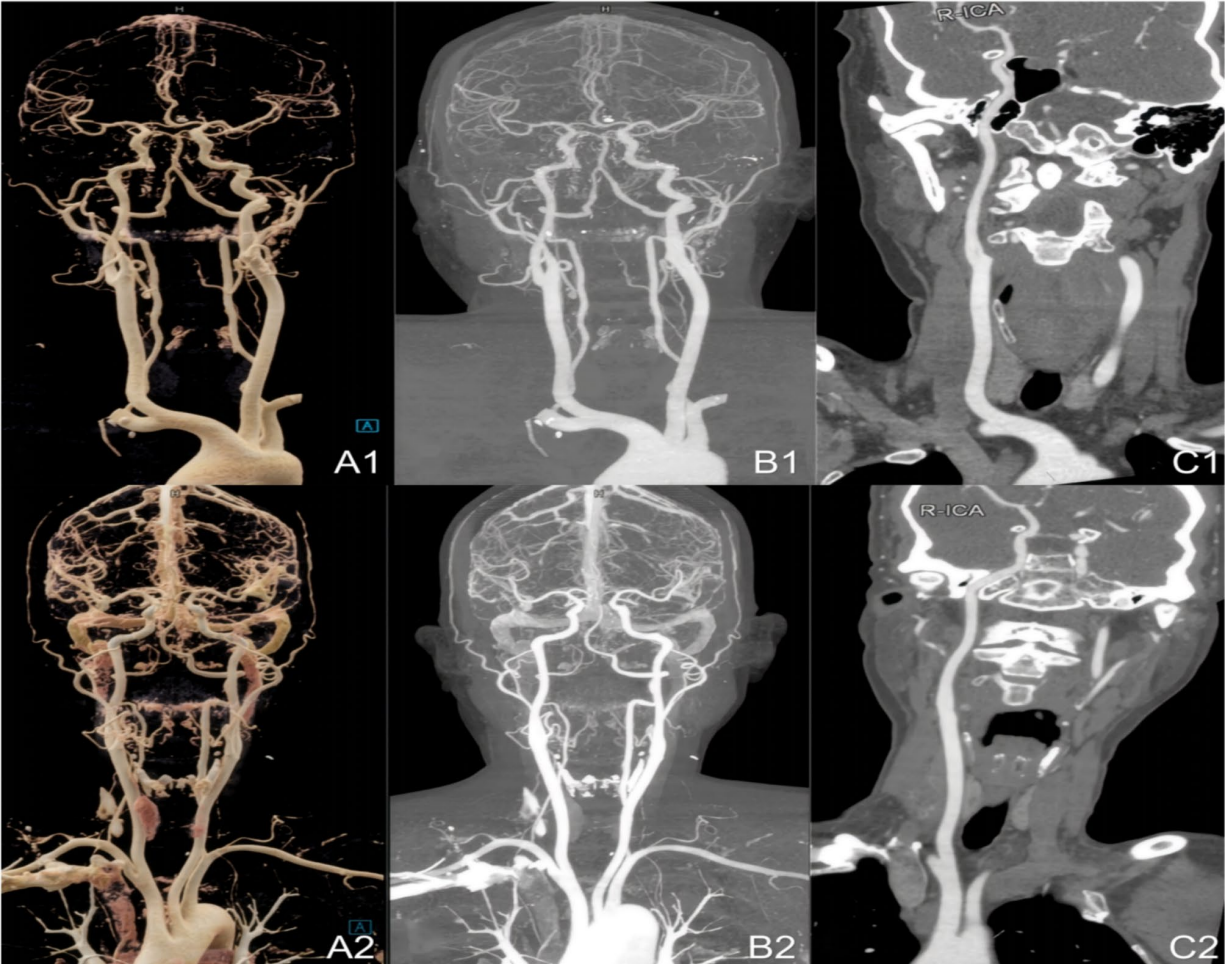
**A1-C1**, a patient with image quality scored at 5 points. **A2-C2**, a patient with image quality scored at 5 points. **A**: volume rendering technique (VRT) image; **B**: maximum intensity projection (MIP) image; **C**: curved planar reformation (CPR) image

Further analysis on the differences in residual contrast agent and artifacts in the right subclavian vein between the two groups revealed that Group A had 60% of images graded as 1, 33% as grade 2, and 7% as grade 3 in the first assessment, with a slight shift in the second assessment (56% for grade 1, 37% for grade 2, and 7% for grade 3). In contrast, Group B had 26.5% of images graded as 1, 58.8% as grade 2, and 14.7% as grade 3 in both assessments, with a slight variation in grade 3 images to 16.7% in the second assessment. Compared to Group B, Group A showed a significant increase in the proportion of grade 1 images (37.9% and 34.9%) and a notable decrease in grade 3 images (36.3% and 41.7%), indicating a significant reduction in residual contrast agent and artifact rates in Group A (Table 5, *P* < 0.001). ( Fig. 4)

## Discussion

Head and neck vascular CTA is pivotal for assessing cervical vascular diseases, underscoring the importance of generating high-quality images for clinical use while minimizing adverse effects and the risk of CIN in patients [18]. The risk of CIN is notably higher in patients with preexisting conditions such as renal impairment, congestive heart failure, diabetes, and in older adults [19]. Additionally, with contrast agents being costly, reducing their use also contributes to medical cost savings.

Current research efforts aim to reduce the usage of contrast agents in head and neck vascular CTA by investigating the efficacy of low tube voltage, dual-energy scanning methods, and optimized contrast agent injection protocols. Low tube voltage techniques for head and neck vascular CTA might result in photon starvation artifacts in the shoulder area for patients with higher body mass, potentially lowering the quality of shoulder artery imaging [20]. Furthermore, Zhao et al. have explored dual-energy scanning methods to reconstruct virtual monoenergetic images that could lower the required contrast agent concentration. However, this approach might not be compatible with dual-energy bone removal functions of post-processing workstations, potentially leading to longer post-processing times and reduced efficiency in bone removal [21]. In this study, third-generation dual-energy scanning images were utilized to circumvent diagnostic challenges that might arise from reduced contrast agent usage in some patients, facilitating the reconstruction of low keV images for image enhancement. The enrolled cases had a blending coefficient (M) of 0.5 (equivalent to approximately 120 kVp images), ensuring that the attenuation values within the arterial images of all patients met diagnostic criteria. Moreover, direct bone removal was achievable in a single scan, negating the need for traditional plain scans and subtraction, thereby streamlining the scanning process and lowering radiation exposure.

Achieving optimal contrast enhancement in vascular imaging entails timing the peak enhancement of the contrast agent to coincide directly with its arrival at the target vessel. In practice, however, the contrast agent’s journey to the target vessel is more of a gradual “climbing” process. After the initial injection, the contrast agent begins to trigger the threshold, awaiting a higher concentration to accumulate in the target vessel. Traditionally, contrast agents are administered at a high flow rate of iodine, which, during the scanning of intracranial arteries, can quickly result in the contrast agent flowing back into the veins, affecting the imaging of both intracranial and cervical veins. Moreover, due to the rapid initiation of scanning, the subsequent saline flush may not sufficiently clear the subclavian vein and superior vena cava, leading to retention of some contrast agent in these veins. In this study, for Group A, we employed a variable rate bolus tracking technique that begins with the injection of a contrast agent at a low iodine flow rate and a lowered trigger threshold. This adjustment allows the ROI to achieve the threshold more gradually, initiating the enhanced scanning program more effectively. During the scanning of intracranial arteries, this method enables the contrast agent to pass through the venous system with reduced venous contamination of the arteries. A second stage, utilizing a high iodine flow rate of the contrast agent, is then employed for optimal imaging of the target vessel. Notably, this approach has significantly minimized the residual contrast agent in the right subclavian vein, demonstrating its effectiveness in improving image quality and reducing venous contamination.

This innovative method of adjusting the injection speed while maintaining a constant iodine concentration has shown promising outcomes in diminishing the residual contrast agent in veins and enhancing the image quality of target vessels. By reducing venous contamination, it holds the potential to increase the diagnostic accuracy of head and neck CTA scans. Furthermore, this technique could contribute to lowering overall costs and reducing the risk of CIN.

The variable rate bolus tracking technique utilized in this study presents potential advantages over the dual-flow injection method, which adjusts the iodine flow rate through altering the saline-contrast agent combination ratio. Firstly, it ensures stability of the iodine flow rate by avoiding the mixture of contrast agent and saline, potentially offering greater consistency in iodine delivery. This approach may prevent the variability seen with mixed injections due to differing injection pressures of contrast agents and saline. Secondly, the operation of this technique is relatively simpler. Finally, compatibility with high-pressure injectors could be broader with the variable rate method since not all injectors support the dual-flow technique, potentially restricting its use in certain clinical environments.

This study’s limitations include the exclusion of patients weighing over 90 kg, possibly influencing arterial segment attenuation values, with 6.9% exceeding 500 HU, likely related to the tailored contrast injection protocol based on body weight. The dilution and diffusion effects of contrast agents are minimal in obese patients due to fat content [22]. Arterial enhancement is influenced by a range of factors: CT scan parameters like tube voltage, scan duration, direction, and delay time; patient-related factors including body weight [23], body surface area (BSA), BMI [24], and cardiovascular circulation time; contrast agent characteristics such as injection rate, duration, volume, bolus injection, and saline flush; and other considerations such as gender, age, venous access, kidney and liver function [25]. Although Group A showed lower intracranial venous sinus attenuation than Group B, the post-processing time for both groups wasn’t detailed, nor were differences between keV levels explored. Future research will delve into these aspects, including the impact of various factors on arterial enhancement, analyzing post-processing time, and examining image differences across keV levels. No CIN occurred in both groups, which may be related to the small sample size of our patients and the better preoperative renal function of the enrolled patients.

Although research on variable-rate bolus tracking techniques is still in its preliminary stages, we have gained clinical application experience. We believe it is necessary to conduct a retrospective analysis of cases where this technique was applied in our center to explore its effects and efficacy in improving imaging quality and ensuring patient safety. This paper primarily uses a retrospective analysis method. Despite its lower statistical validity compared to prospective studies, we believe our findings have clinical significance, hence reporting our retrospective study results. Additionally, being a single-center retrospective study, these findings will seek validation in multi-center studies across different regions.

## Conclusion

Employing variable-rate bolus tracking technology alongside third-generation dual-source CT dual-energy scanning in head and neck vascular CTA leads to high-quality images and markedly lowers the required contrast agent dosage. This method enhances the attenuation contrast between intracranial arteries and veins and minimizes the residue and artifacts of high-density contrast agents in the right subclavian vein.

## Data Availability

The datasets used and analyzed during the current study are available from the corresponding author on reasonable request.
